# Cellular Senescence and the Kidney: Potential Therapeutic Targets and Tools

**DOI:** 10.3389/fphar.2019.00770

**Published:** 2019-07-12

**Authors:** Sebastian N. Knoppert, Floris A. Valentijn, Tri Q. Nguyen, Roel Goldschmeding, Lucas L. Falke

**Affiliations:** ^1^Department of Pathology, University Medical Center Utrecht, Utrecht, Netherlands; ^2^Department of Internal Medicine, Diakonessenhuis, University Medical Center Utrecht, Utrecht, Netherlands

**Keywords:** senescence, chronic kidney disease, kidney fibrosis, senolytics, targeted therapy

## Abstract

Chronic kidney disease (CKD) is an increasing health burden (affecting approximately 13.4% of the population). Currently, no curative treatment options are available and treatment is focused on limiting the disease progression. The accumulation of senescent cells has been implicated in the development of kidney fibrosis by limiting tissue rejuvenation and through the secretion of pro-fibrotic and pro-inflammatory mediators termed as the senescence-associated secretory phenotype. The clearance of senescent cells in aging models results in improved kidney function, which shows promise for the options of targeting senescent cells in CKD. There are several approaches for the development of “senotherapies”, the most rigorous of which is the elimination of senescent cells by the so-called senolytic drugs either newly developed or repurposed for off-target effects in terms of selectively inducing apoptosis in senescent cells. Several chemotherapeutics and checkpoint inhibitors currently used in daily oncological practice show senolytic properties. However, the applicability of such senolytic compounds for the treatment of renal diseases has hardly been investigated. A serious concern is that systemic side effects will limit the use of senolytics for kidney fibrosis. Specifically targeting senescent cells and/or targeted drug delivery to the kidney might circumvent these side effects. In this review, we discuss the connection between CKD and senescence, the pharmacological options for targeting senescent cells, and the means to specifically target the kidney.

## Introduction of Renal Disease and Senescence

### Renal Disease as a Major Individual and Global Burden

Chronic kidney disease (CKD) is defined by the persistent loss of kidney function and currently affects approximately 13.4% of the global population (Hill et al., 2016; Jager and Fraser, 2017). The progressive nature of CKD often leads to end-stage renal disease (ESRD), requiring renal replacement therapy. To date, there are no curative therapeutic options for CKD/ESRD. Survival on dialysis remains poor (Collins et al., 2010), and there is a global shortage of kidney donors, because (among other reasons) the kidney transplant 10-year graft survival rate is only 60% at best due to rejection and the progressive loss of graft function (Matas et al., 2015). Furthermore, for transplant recipients, the lifelong use of immunosuppressive therapy is mandatory. Therefore, any progress in the development of therapies that could prevent CKD progression (and ultimately ESRD altogether) could have a major societal impact.

Current therapy consists of treating CKD complications and slowing CKD progression by targeting known risk factors for disease progression, such as salt and protein intake, hypertension, and glomerular hyperfiltration.

An as yet untreatable final common pathway irrespective of the etiology in CKD is kidney fibrosis, characterized histologically by glomerulosclerosis, tubular atrophy, and interstitial fibrosis (Liu, 2011). Numerous compounds directly targeting factors involved in fibrosis driving pathways are currently being studied with varying results [e.g., transforming growth factor-β (TGF-β) signaling pathway inhibitors (pirfenidone and fresolimumab; Meng et al., 2016), anti-CCN2/connective tissue growth factor (CTGF; pamrevlumab; Kok et al., 2014), and tyrosine kinase inhibitors (e.g., nintedanib, gefitinib, and imatinib)]. This approach shows some promise, but clinical trial results vary (Klinkhammer et al., 2017). Apart from the use of the renin-angiotensin-aldosteron pathway interfering agents such as ACE inhibitors or angiotensin receptor blockers to reduce the progressive remodeling of renal parenchyma, no therapeutics addressing pathophysiological mechanisms underlying CKD are used clinically. However, increasing effort is currently put into investigating the efficacy of targeting senescent cells during renal disease.

### Cellular Senescence: General Aspects

During life, cells are unavoidably exposed to various stresses that potentially cause DNA damage [e.g., reactive oxygen species (ROS), ionizing/ultraviolet (UV) radiation, sheer stress, chemical injury, or replicative stress]. To ensure integrity, DNA is checked during cell cycle progression and, when needed and possible, repaired. Major DNA integrity checks occur during the G1/S or G2/M transition phases of the cell cycle (Moonen et al., 2018). When DNA injury is irreparable, either apoptosis or a permanent inhibition of cell cycle progression occurs despite growth factor stimulation. The latter situation/condition is also known as cellular senescence (Campisi and d’Adda di Fagagna, 2007). Replicative senescence is triggered by telomere attrition resulting from repeated cell division, whereas stress-induced premature senescence is due to oxidative and genotoxic stresses (Campisi and d’Adda di Fagagna, 2007; Toussaint et al., 2000). The exact mechanism of DNA damage detection, cell cycle checkpoint control, and the mechanism of renal senescence are expertly reviewed elsewhere (Campisi and d’Adda di Fagagna, 2007; Ashwell and Zabludoff, 2008; Sturmlechner et al., 2017; Singh and Wu, 2019).

**Figure 1:**
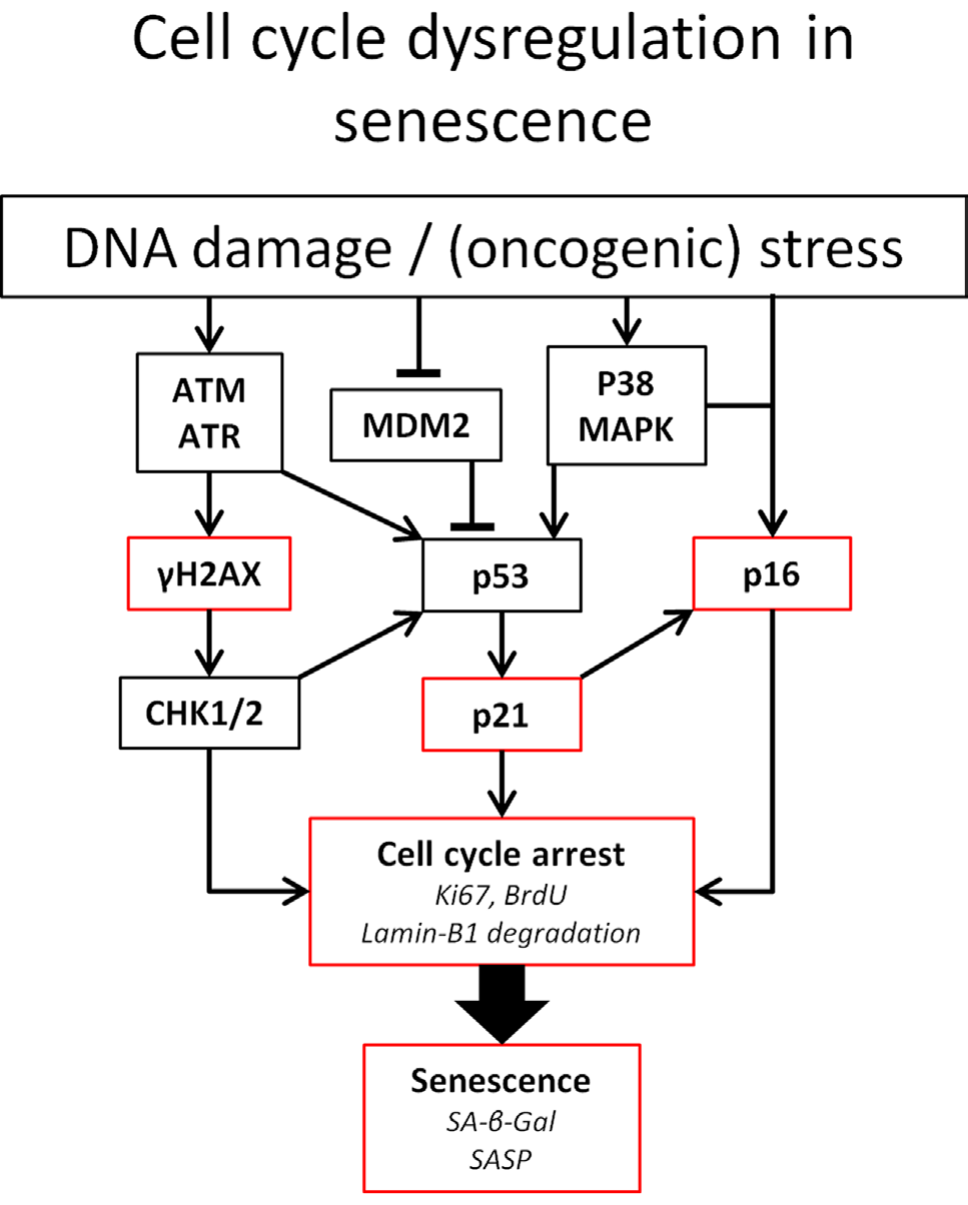
Pathways involved in cell cycle arrest. Established means to identify senescent cells are indicated in red. Ataxia telangiectasia mutated (ATM) and ATM-Rad3-related (ATR) kinases play a central role in DNA damage detection and response. Both proteins rapidly phosphorylase histone 2AX. Furthermore, ATM and ATR can both lead to the phosphorylation of the tumor suppressor p53(pSer15) upon DNA damage. Furthermore, ATM phosphorylates the checkpoint kinases Chk1 and Chk2. Chk2 directly phosphorylates p53 at pSer20, leading to the expression of the CKI p21. Phosphorylation of p21 inhibits CDK, leading to the hypophosphorylation of retinoblastoma tumor suppressor (Rb). This enables Rb to bind to E2F, inhibiting cell cycle progression. Likewise, p53 activity can be increased by p38 MAPK activity, induced by ROS, or by binding of p14^ARF^ to murine double minute 2 (MDM2) preventing degradation of p53 (Elledge and Zhou, 2000; Hirao et al., 2002; Fischer et al., 2016). In addition to p53, the accumulation of the tumor suppressor p16^Ink4a^ also leads to cell cycle arrest *via* the inhibition of CDK4/CDK6 and subsequent hypophosphorylation of Rb (Moonen et al., 2018). Abbreviations: BrdU, bromodeoxyuridine.

As depicted in **Figure 1**, p53 and p16 are key regulators of cell fate in the setting of DNA damage. Particularly, the level of p53 expression determines whether an arrested cell i) continues replication upon DNA damage resolution, ii) becomes senescent, or iii) goes into apoptosis (Purvis et al., 2012). The expression of either pro-cell cycle arrest or pro-apoptotic molecules shows a linear increase with p53. p53-mediated apoptosis induction is threshold dependent and low levels of p53 can be sufficient to induce cell cycle arrest (Kracikova et al., 2013).

Senescence is an important driver of fibrosis. Senescent cells acquire a senescence-associated secretory phenotype (SASP) characterized by the expression and secretion of pro-fibrotic and pro-inflammatory factors. These SASP factors act upon neighboring healthy cells in a paracrine fashion, thereby driving the progression of fibrosis in CKD (Coppe et al., 2010; de Keizer, 2017). Senescent cells are mostly cleared by the immune system but accumulate during the aging process (Hoenicke and Zender, 2012).

Recent evidence suggests that senescence may play a key role in CKD progression (Valentijn et al., 2018). As many factors associated with SASP are known to induce fibrosis in the kidney [e.g., TGF-β, CCN2 (also known as CTGF), interleukin (IL)-1, and IL-6] (Wang et al., 2017), targeting senescence might prove an effective alternative strategy for CKD treatment. This review aims to 1) provide a concise description of the pathophysiology of cellular senescence in the kidney and 2) discuss the various potential intervention points within the senescence network.

### Pro-Survival Pathways in Senescence

Apoptosis resistance is an important characteristic of senescent cells and the most widely and intensely explored target for therapeutic intervention. Telomere attrition, DNA damage, and other stressors typically induce cells to up-regulate pro-apoptotic factors, the effect of which is counteracted by the simultaneous increase of anti-apoptotic factors to prevent their premature cell loss. Thus, shifting the balance toward the dominance of anti-apoptotic factors constitutes the “Achilles’ heel” of senescent cells that circumvent apoptosis (Wang, 1995; Sasaki et al., 2001; Sagiv et al., 2013; Zhu et al., 2015).

As a result, senescent cells become even more resistant to intrinsic and extrinsic pro-apoptotic stimuli than non-senescent cells, as exemplified by higher survival and less apoptosis after tumor necrosis factor-α (TNF-α) treatment and UV irradiation (Yosef et al., 2016). An expert review on the apoptotic balance in cellular senescence is provided by Childs et al. (2014).

The activation of mitochondrial anti-apoptotic B-cell lymphoma 2 (BCL-2) family members (BCL-2, BCL-W, BCL-X_L_, MCL-1, and A1) has been identified as the central molecular mechanism by which senescent cells resist apoptosis. Knockout (KO) of a combination of BCL-W, BCL-X_L_, and BCL-2 leads to the reduced viability of senescent cells, showing that these cells depend on the (over)expression of anti-apoptotic factors to prevent “spontaneous” apoptosis (Chang et al., 2016; Yosef et al., 2016).

Tied to their dependence on BCL-2 protein family members, senescent cells are reliant on pro-survival pathways involving the p53-p21-serpine and phosphoinositide 3-kinase (PI3K)/AKT pathways. As such, the ephrin-dependent receptor ligands ephrin B1 and B3 and plasminogen activator inhibitor-1 (PAI-1) are also implicated in pro-survival signaling in senescent cells (Zhu et al., 2015).

The exact interplay of pro- and anti-apoptotic proteins is complex (Fuchs and Steller, 2015). In case of irreversible DNA damage, the DNA damage response (DDR) mediates apoptosis *via* the activation of pro-apoptotic proteins such as phorbol-12-myristate-13-acetate-induced protein 1 (also known as NOXA) and the BCL-2 homology domain 3 (BH3)-only protein Bim and the activation of p53 up-regulated modulator of apoptosis (PUMA) that binds and inhibits multiple anti-apoptotic BCL family members (O’Connor et al., 1998; Chen et al., 2005).

Interestingly, Baar et al. (2017)found that the pro-apoptotic PUMA and BIM were up-regulated in radiation-induced senescent IMR90 fibroblasts, whereas the anti-apoptotic BCL-2 was reduced in senescent cells, making these cells, in theory, more susceptible to apoptosis. However, the reduction of BCL-2 in irradiated fibroblasts is not consistently seen and the expression of pro- and anti-apoptotic proteins may vary depending on the cell type (Zhu et al., 2016), which could explain the observed differences in response to therapies targeting specific apoptotic pathways.

### Detecting Cellular Senescence

To date, a definitive sensitive *and* specific marker for cellular senescence has not been identified. Hallmarks of senescent cells are their resistance to apoptosis and phenotypic changes, including altered morphology with large flattened cell bodies. Another interesting characteristic of senescent cells is the increased lysosomal content resulting in lysosomal β-galactosidase (β-gal) activity (also known as senescence-associated β-Gal or SA-β-Gal) being readily detectible at the enzymatically suboptimal and relatively high pH of 6.0 (Lee et al., 2006). However, SA-β-Gal is typically also increased in non-senescent, high-density, and confluent cell cultures, which limits its applicability as a stand-alone marker for the detection of senescent cells. Furthermore, senescent cells can be detected based on the activity in the pathways leading to cell cycle arrest (**Figure 1**), e.g., increased phosphorylation of histone H2AX (γ-H2AX) and accumulation of cyclin-dependent kinase (CDK) inhibitors (CKIs) such as tumor protein p53 (TP53 or p53), p21^Cip1^ (p21), and p16^Ink4a^ (p16), and by the increase of senescence-associated heterochromatin foci. The importance of p16 is illustrated by the beneficial effect the elimination of p16-expressing cells has on age-related deterioration (Baker et al., 2011). In contrast, senescent cells stop expressing proliferation markers (e.g., Ki-67) and produce a senescence-associated secretome (SASP; see SASP in CKD).

Another particularly interesting feature of senescent cells is the loss of the structural nuclear lamina component lamin B1. The importance of reduced nuclear lamina integrity is underlined by the progeria phenotype of the Hutchinson-Gilford syndrome caused by the loss-of-function mutations in the lamin A gene (De Sandre-Giovannoli et al., 2003). In apoptotic cells, lamin B1 is degraded by caspases, whereas, in senescent cells, lamin B1 decrease results from the reduced lamin B1 mRNA stability. Of note, no decrease in lamin B1 is seen in quiescent cells (Freund et al., 2012).

Combinations of several of the markers mentioned above have been used to identify senescent cells *in vitro* and *in vivo* (Myrianthopoulos, 2018). **Table 1** shows the most frequently used markers of senescence in mammal studies.

**Table 1:** Markers used to identify senescent cells.

Marker	Reference
Degradation of lamin B1	(Shimi et al., 2011; Freund et al., 2012; Sadaie et al., 2013; Hernandez-Segura et al., 2017)
SA-β-gal at pH 6.0	(Dimri et al., 1995; Lee et al., 2006)
Lipofuscin accumulation	(von Zglinicki et al., 1995; Rizou et al., 2019)
HMGB1 relocalization	(Davalos et al., 2013)
Senescence-associated heterochromatin foci	(Narita et al., 2003)
Increased SIRT2 (NAD^+^-dependent HDAC III class enzyme) expression	(Anwar et al., 2016)
(phosphorylated) p38 MAPK	(Wang et al., 2002)
p16^Ink4a^ expression	(Alcorta et al., 1996; Stein et al., 1999)
p21^WAF/CIP1^ expression	(Alcorta et al., 1996; Stein et al., 1999; Lopez-Otin et al., 2013)
Flattened and enlarged phenotype	(Serrano et al., 1997)
Absence of proliferation markers (Ki-67, BrdU, EdU)	(Lawless et al., 2010; Hernandez-Segura et al., 2018)
γ-H2AX foci	(Lawless et al., 2010)
PAI-1 overexpression	(Goldstein et al., 1994)
Expression of SASP factors (e.g., IL-1α, IL-1β, IL-6, IL-8, MCP-1, and CCN2)	(Kim et al., 2004; Freund et al., 2010)
Expression of Dec1 [class E basic helix-loop-helix protein 40 (BHLHE40)]	(Collado et al., 2005)
Expression of DcR2 [TNF receptor superfamily member 10D (TNFRSF10D)]	(Collado et al., 2005)

## Cellular Senescence in the Aging and Injured Kidney

### Aging

Aging is associated with the decline of kidney function. During aging, increased renal p16 expression is most notably seen in tubular epithelium and to a lesser extent in glomerular (podocytes and parietal epithelium) and interstitial cells. Changes in p16 were more pronounced in the cortex compared to the medulla (Melk et al., 2004; Sis et al., 2007). In rodents, the amount of senescent proximal tubular cells increases with age, whereas no increase of senescent cells is seen in the glomeruli. Renal tubular cell senescence correlates with tubular atrophy, interstitial fibrosis, and glomerulosclerosis (Verzola et al., 2008; Liu et al., 2012). Furthermore, the removal of senescent tubular cells leads to decreased glomerulosclerosis (Baker et al., 2016). Along with increased tubular senescence, an increase in p21 and TGF-β1 expression is measured in the tubulo-interstitium (Ding et al., 2001). In aging mice kidney, an age-dependent increase is seen in cortical p21 mRNA expression and an increase in p21 plasma concentration, but no increase in urinary p21 excretion was observed (Johnson and Zager, 2018).

### Injury

Acute damage of tubular epithelial cells [e.g., transplantation-associated ischemia-reperfusion injury (IRI) or acute kidney injury (AKI)] leads to DNA damage and induces an intrinsic DDR. Proximal tubular epithelial cells are especially susceptible to injury (ischemia or toxic injury) (Bonventre et al., 2011). Upon injury, a substantial number of tubular cells undergo apoptosis or are shed *via* the urine. Due to the regenerative capacity of tubular cells, lost cells are largely replaced *via* the proliferation of neighboring tubular epithelium (Duffield et al., 2005; Humphreys et al., 2008). However, prolonged or repeated renal injury leads to maladaptive repair (i.e., inflammation, myofibroblast accumulation, fibrosis, and vascular rarefaction) leading to CKD. AKI predisposes kidneys to CKD development with age or after a second hit. Alternatively, CKD patients are more prone to AKI development upon injury (Chawla and Kimmel, 2012). A possible explanation lies in the accumulation of senescent cells during aging and post-injury, given that after AKI the senescent cell burden slowly accumulates over time (Jin et al., 2019).

The severity of allograft nephropathy, diabetic nephropathy, and IgA nephropathy is associated with the level of senescence (Joosten et al., 2003; Verzola et al., 2008; Liu et al., 2012). Additionally, the level of senescence before kidney transplantation could predict the outcome in terms of graft function (McGlynn et al., 2009), suggesting that targeting senescent cells could be an effective therapeutic intervention in kidney disease.

As proof of principle for the therapeutic potential of targeting senescence, several studies were conducted showing the attenuation of functional decline and fibrosis. Small interfering RNA-based p53 inhibition after IRI reduces cellular senescence and is associated with the attenuation of kidney fibrosis in rats (Molitoris et al., 2009). This phenomenon is thought to be in part related to the effects of G2/M cell cycle-arrested tubular cells, which have been shown to produce excessive amounts of TGF-β and CCN2 as part of SASP (Yang et al., 2010; Bonventre, 2014). These factors are also associated with kidney fibrosis (Phanish et al., 2010).

Renal diseases such as IgA nephropathy and lupus nephritis associate with increased senescent cell burden (Liu et al., 2012; Yang et al., 2018), but diabetes mellitus is the most studied disease regarding cellular senescence in the kidney.

Exposure of the kidney to high blood glucose levels in patients with type 2 diabetes significantly increases their renal senescent cell burden. This induction of senescence is specifically seen in tubular epithelium cells and podocytes (Verzola et al., 2008). Only 7 days of (streptozotocin-induced) hyperglycemia already increases senescent cell burden in the mouse kidney and contributes to the acquisition of SASP (Prattichizzo et al., 2018). The accumulation of senescent cells is also seen in hyperglycemic rats, where the most pronounced effect is seen in the cortical tubules of the kidney at 10 days (Satriano et al., 2010). Interestingly, the clearance of senescent cells improves glucose homeostasis and insulin sensitivity in a mouse model of obesity-induced metabolic dysfunction. The clearance of senescent cells in this model also resulted in improved renal podocyte function and reduced microalbuminuria (Palmer et al., 2019).

### Benefits of Senescence During Kidney Injury

Contrary to the general opinion backed by evidence, some contradictory evidence exists regarding the beneficial role of senescence during renal injury. One study shows in a short-term model of unilateral ureter obstruction (UUO), a robust model for kidney atrophy and fibrosis, that cell cycle arrest might be favorable in the acute phase of injury. p16-KO mice show increased kidney damage and fibrosis compared to wild-type littermates (Wolstein et al., 2010). Similar results were seen in a renal ischemia-reperfusion mice model, where senescence induction with a CDK4 and CDK6 inhibitor improved serum creatinine and blood urea nitrogen (DiRocco et al., 2014). This is consistent with previous evidence in other tissues that the effects of SASP secretion (e.g., stemness induction) might have benefits in the handling of acute injuries, whereas prolonged SASP exposure results in impaired tissue repair (de Keizer, 2017).

### SASP in CKD

In the long term, senescent cells may impair tissue function and the rejuvenation of their environment. Underlying the detrimental effects of prolonged cell cycle arrest is SASP. The precise SASP composition varies between cell types and mode of senescence induction and depends on time after senescence induction (Hernandez-Segura et al., 2017). Furthermore, all known SASP factors are also implicated in other non-senescent cell conditions (Coppe et al., 2010). In addition to the previously mentioned pro-fibrotic factors, SASP consists of an array of inflammatory chemokines and cytokines, allowing cross-talk between senescent cells and neighboring cells and facilitating the detection and elimination of senescent cells by the immune system (i.e., immune surveillance) (Kang et al., 2011). See **Table 2**. For a more extensive list, see Freund et al. (2010) and Wang et al. (2017). Although SASP has detrimental effects in the long term, the short-term effects of SASP can be beneficial (e.g., during embryogenesis and wound healing) (Storer et al., 2013; Demaria et al., 2014). Moreover, the secretion of SASP components such as vascular endothelial growth factor (VEGF) and fibroblast growth factor 2 promote tissue repair during kidney injury (Bonventre, 2014; Yan et al., 2017).

**Table 2:** SASP factors associated with inflammation or fibrosis.

Pro-inflammatory	Pro-fibrotic
IL-1α	TGF-β
IL-1β	CNN2 (CTGF)
IL-6	VEGF
IL-7	PDGF
IL-8	
MCP-1	
MCP-2	
MIP-1α	
IFN-γ	
TNF-α	

SASP factors can influence both the innate and adaptive immune responses in either promoting clearance by the immune system or causing immunosuppression, thereby promoting the elimination or persistence of senescent cells, respectively (Burton and Stolzing, 2018). An impaired immune system (due to aging, disease, or immunosuppressive therapy) may cause some senescent cells to evade elimination and maintain an SASP secretome (Lujambio, 2016). Exposure to dedifferentiating SASP factors (primarily IL-1β and IL-6) induces a state of pluripotency in neighboring cells, leaving these cells unable to differentiate (“stem lock”) (Brady et al., 2013; Pietras et al., 2016; de Keizer, 2017). Thus, through SASP, senescent cells can harm healthy neighboring cells and also block the renewal of lost or damaged cells. This paracrine effect of senescent cells is exemplified by Xu et al. (2018), showing that intraperitoneal injection of senescent preadipocytes in mice resulted in increased physical dysfunction and increased cellular senescence in the recipient tissues along with increased SASP factors. Furthermore, da Silva et al. (2019) showed that xenotransplantation of senescent human fibroblasts to immunodeficient mice led to an increased expression of senescence markers in the surrounding tissues.

The ability of the kidney to acquire a SASP has been illustrated by Prattichizzo et al. (2018) and Zhang et al. (2017). Both in mice with a hyperglycemic milieu and in mice with an accelerated aging phenotype, the kidneys showed increased transcription of key SASP mRNAs (most notably IL-1β and IL-6) and increased amounts of SASP proteins (namely IL-1β) (Zhang et al., 2017; Prattichizzo et al., 2018). Interestingly, Prattichizzo et al. (2018) showed that endothelial cells and macrophages are an important source of SASP factors in the kidney in a hyperglycemic milieu. Recently, Yao et al. (2019) showed that knockdown of fibroblast-specific PAI-1 (a well-established SASP factor) reduced renal fibrosis after kidney injury. The secretion of SASP factors contribute to the formation of kidney fibrosis after kidney injury. Thus, multiple cell types in the kidney are implicated in the formation of kidney fibrosis.

Although all known constituents of SASP can also be induced independent of cellular senescence, the overlap between SASP and the factors increased in CKD models and patients is quite remarkable (Wang et al., 2017). For example, in a mouse model of kidney fibrosis after IRI, increased expression of MCP-1 (also known as CCL2) and TNF-α is observed (Clements et al., 2013). Increased levels of MCP-1, epidermal growth factor, VEGF, IL-6, and IL-1α are also detected early in the peripheral blood of patients with CKD progression (Perlman et al., 2015). In addition, spot-urine analysis in CKD patients showed the presence of MCP-1, IL-8, and TGF-β1, whereas only MCP-1 could be detected in healthy control urine. However, increased plasma and urinary concentration of MCP-1, IL-8, and TGF-β1 did not correlate with increase of CKD stage (Vianna et al., 2013).

SASP is regulated by several transcription factors, the main regulators being nuclear factor-κB (NF-κB) and C/EBPβ (Orjalo et al., 2009). Aside from SASP, NF-κB signaling mediates the balance between apoptosis and autophagy (Salminen et al., 2012). Autophagy is seen as both pro-senescent (mainly in oncogene induced) and anti-senescent (less defective mitochondria and ROS) (Kwon et al., 2017). Another regulatory protein involved in SASP secretion are sirtuins. Sirtuin activity regulates DNA repair, apoptosis, inflammation control, and antioxidative defense. Sirtuins (mainly SIRT1) inhibits NF-κB, decreasing SASP secretion (Salminen et al., 2012; Grabowska et al., 2017). Mammalian target of rapamycin (mTOR) serves as an important mediator of SASP secretion. During senescence, autolysosomes and mTOR accumulate in a specific compartment called the TOR autophagy spatial coupling compartment (TASCC). Recently, it was discovered that the TASCC is associated with the secretion of TGF-β and CCN2 in tubular epithelium cells and is associated with increased fibrosis after kidney injury (Narita et al., 2011; Canaud et al., 2019). Remarkably, CCN2 can by itself increase AKT kinase activity and promote senescence in epithelial cells and thus play a role in the paracrine spread (furtherance) of senescence driving CKD (Jang et al., 2017).

### Kidney Fibrosis

When epithelial cells become senescent, this leads to maladaptive repair and contributes to the progression of kidney fibrosis (Ferenbach and Bonventre, 2015). Furthermore, senescent cells contribute to a pro-fibrotic milieu through their SASP (Tchkonia et al., 2013), and senescence markers correlate with the amount of kidney fibrosis in mice (Clements et al., 2013). Additionally, the importance of senescence in the process of kidney fibrosis is illustrated by the decrease of fibrosis after IRI in mice lacking p16 expression either due to the loss of the INK4a locus or the short hairpin RNA-mediated silencing of p16 (Braun et al., 2012; Luo et al., 2018). Although these studies mostly pertain animals, evidence suggests that senescence is also implicated in human kidney fibrosis. In human transplant biopsies, the senescent cell burden correlates with the amount of fibrosis and tubular atrophy, and the senescent cell burden can predict transplant kidney function (Ferlicot et al., 2003; Melk et al., 2004; McGlynn et al., 2009; Gunther et al., 2017).

### Malignancy

The role of cellular senescence in tumor suppression is well established (Sharpless et al., 2004). Mitosis of cells containing DNA damage or the activation of oncogenes can be inhibited by senescence mechanisms (Bielak-Zmijewska et al., 2018). Thus, inactivating senescent pathways makes these cells more susceptible to malignant transformation. For example, p21-deficient mice with reduced senescence show an increased susceptibility for tumor development (Martin-Caballero et al., 2001). Furthermore, signs of loss of this tumor-suppressive mechanism in renal cell carcinoma are associated with a more proliferative phenotype and a higher tumor grade (Macher-Goeppinger et al., 2013).

Aside from preventing tumorigenesis, cellular senescence can also reduce the proliferative ability of malignancies (Zeng et al., 2018). This is used in clinical practice by senescence induction by (a combination of) chemotherapy, radiation therapy, or CDK4 and CDK6 inhibitors (Shah et al., 2018; Zeng et al., 2018). However, systemic chemotherapy also induces senescence in otherwise healthy tissues, leading to local and systemic inflammation and detrimental short- and long-term effects. These effects have been implicated in increased cancer recurrence and cancer metastasis (Demaria et al., 2017). Furthermore, SASP factors such as CCN2 promote chemotherapy resistance of cancer cells (Sun et al., 2012; Yang et al., 2016; Zeng et al., 2017).

Immunosurveillance is an important mechanism of senescent cell clearance (Sagiv and Krizhanovsky, 2013), and in line with this, chronic immunosuppression might contribute to the accumulation of senescent cells after kidney transplantation. Clinically, this is supported by the observation that calcineurin inhibition leads to an altered natural killer cell phenotype (a major contributor to immunosurveillance) and thus reduced immunosurveillance and persistence of senescent cells (Hoffmann et al., 2015). In addition to the loss of kidney function resulting from the deleterious effect of SASP on the microenvironment, evidence suggests that failure of senescent cell elimination might also be oncogenic (Coppe et al., 2010).

Thus, cellular senescence can be both beneficial and detrimental in the prevention and treatment of malignancies (Zhang et al., 2019).

## Senotherapy

Eliminating senescent cells through transgenic depletion and pharmaceutical inhibition reduces kidney dysfunction and long-term kidney injury in experimental models of kidney damage, obesity-induced metabolic dysfunction, and during aging (Valentijn et al., 2018). These promising results have spurred interest in the development of clinically applicable therapeutic compounds that target senescence-associated pathways.

Eliminating senescent cells (dubbed as senolysis) is just one of the various potential interventional approaches to target the adverse effects of cellular senescence (so-called “senotherapy”), including the prevention of senescence, modulation of SASP (termed senomorphics), and stimulation of immune system-mediated clearance of senescent cells (reviewed by Kim and Kim, 2019).

### Senomorphics and Prevention of Senescence

Several therapeutic options have been shown to modulate SASP and prevent senescence by interfering with senescent-associated intracellular pathways. These therapeutic agents have not been shown to specifically induce apoptosis in senescent cells, as opposed to senolytics. For instance, glucocorticoids suppress SASP and reduce IL-6 in senescent HCA2 medium (Laberge et al., 2012). The inhibition of the JAK/STAT pathway by JAK inhibitor 1 reduces the transcription of senescent preadipocyte SASP factors such as IL-6, IL-8, IP-10, CXCL-1, MCP-1, and MCP-3 (also known as CCL7) (Xu et al., 2015). The p38 inhibitors UR-13756 and BIRB 796 both decreased IL-6 suppression (Alimbetov et al., 2016). Several other well-established senomorphic and senescence preventing agents are discussed below.

#### Metformin

Metformin, a biguanide used in the treatment of type 2 diabetes, shows promise as an anti-aging agent as reviewed by Kanigur Sultuybek et al. (2019). The decrease in blood glucose by metformin is mainly achieved by the reduction of gluconeogenesis and increased glucagon signaling. However, metformin has several other effects, most notably the activation of AMP-activated protein kinase (AMPK) and the down-regulation of the mTOR pathway (Pernicova and Korbonits, 2014). In 2016, the Food and Drug Administration (FDA) revised the guidelines for metformin treatment in patients with CKD, now allowing patients with CKD to be treated with metformin. This is a very promising step given the renal protective abilities of metformin in patients with diabetic nephropathy and even non-diabetic kidney disease, as reviewed by Ravindran et al. (2017). Although the precise mechanism by which metformin attenuates age-related disease is unknown, it is suggested that metformin inhibits NF-κB signaling, thus inhibiting SASP (Moiseeva et al., 2013; Noren Hooten et al., 2016; Kanigur Sultuybek et al., 2019). An *in vitro* study has shown that metformin treatment delayed senescence in human diploid fibroblasts and human mesenchymal stem cells (MSCs), probably through increasing GPx7 and Nrf2 (Fang et al., 2018). Furthermore, metformin reduces ROS formation, γ-H2AX foci, and ATM, in turn reducing the formation of senescent cells (Halicka et al., 2011; Park and Shin, 2017). Besides age-related disease, metformin has a beneficial effect in the treatment of cancer by inhibiting metabolism, reducing protumorigenic signaling through NF-κB inhibition, and reducing stemness in cancer cells that escape oncogene-induced senescence (Deschenes-Simard et al., 2019).

#### Rapamycin

Rapamycin, an mTOR inhibitor, can potently reduce oxidative injury by inhibiting protein synthesis and stimulating intracellular repair and autophagy. Rapamycin (and its derivatives) are currently used as a potent immunosuppressive drug after solid organ transplantation (Nguyen et al., 2019) and are used (in higher concentrations) for their oncolytic properties in advanced renal cell carcinoma (Boni et al., 2009).

In UUO mice, treatment with rapamycin reduces the fibrotic response in the kidney (Falke et al., 2015) and rapamycin treatment increases the lifespan of middle-aged mice (Bitto et al., 2016). *In vitro* experiments with rapamycin resulted in an increased proliferation of senescent cells (Demidenko et al., 2009). As such, this drug is suggested to alleviate the senescent burden and could explain the positive effects seen in the mice models. However, to achieve this effect, even higher concentrations are needed than the concentrations used for the oncolytic properties and this may limit the clinical translation (Kaeberlein, 2014).

Senescent cells, like cancer cells, are highly metabolically active and thus are susceptible to the inhibition of protein synthesis and cell growth by rapamycin *via* the inhibition of both mTOR complexes 1 and 2 (Li et al., 2014). Thus, mTOR inhibition can both induce apoptosis in malignant cells and reduce senescent cell burden.

Although it is not entirely clear whether especially senescent tumor cells are sensitive to the oncolytic activity of rapamycin, generally speaking, senescent as well as non-senescent cells with high metabolic activity might share increased dependence on the mTOR pathway for their survival. mTOR inhibition has shown beneficial effects in the setting of organ transplantation, cancer, and senescence, but the inhibition of mTOR can also lead to serious side effects (Nguyen et al., 2019). These side effects include insulin resistance, glomerular dysfunction, dyslipidemia, hematologic side effects (anemia, leucopenia, and thrombocytopenia), mucositis, pneumonitis, lymphedema, angioedema, and osteonecrosis. This wide variety of side effects likely reflects the broad involvement of the mTOR pathway in cell and tissue homeostasis.

#### Niacin and Resveratrol

Another option to reduce the development of cellular senescence is *via* sirtuin activation. As mentioned above, SIRT1 inhibits NF-κB signaling, which is a key transcriptional regulator of SASP, but sirtuins also alleviate cell cycle arrest by regulation of p53, NF-κB, STAT, FOXO1, and FOXO3 (Wakino et al., 2015). A decrease in sirtuin activation is implicated in the reduction of kidney function with age (Ugur et al., 2015) and increased activation of SIRT1 attenuates high glucose-induced kidney mesangial hypertrophy (Zhuo et al., 2011). Increased sirtuin activation can be achieved *via* dietary supplementation of NAD^+^ with niacin, such as nicotinamide riboside (Rajman et al., 2018). SIRT activation can also be achieved by resveratrol, although the beneficial effect of resveratrol on senescence markers is seen in analogy to resveratrol without SIRT1 activation (Latorre et al., 2017). Although nicotinamide riboside and resveratrol have a positive effect on the health and lifespan of animals (Gambini et al., 2015; Rajman et al., 2018), they do not induce a senolytic effect (Latorre et al., 2017; Grezella et al., 2018).

### Senolytics

The removal of senescent cells with so-called “senolytics” may be the most feasible and most attractive approach for clinical application, as the prevention of senescence and modulation of SASP would require chronic treatment with prolonged exposure to therapeutics.

Senolytic agents are a class of small molecules that can selectively kill senescent cells that participate in senescence-associated pathways by interfering with anti- and pro-survival signaling (Zhu et al., 2015).

Because of the same reliance on anti-apoptotic signaling of tumor cells as seen in senescence, similar therapeutic strategies are being explored in oncology as can be used for effective/specific senescence depletion, and the kidney community may well benefit from the yield of such studies.

Several senolytic drugs are already in clinical use or in advanced phases of anti-tumor drug development, providing relevant information concerning efficacy and safety that may be extrapolated at least in part to non-oncological kidney diseases.

**Figure 2:**
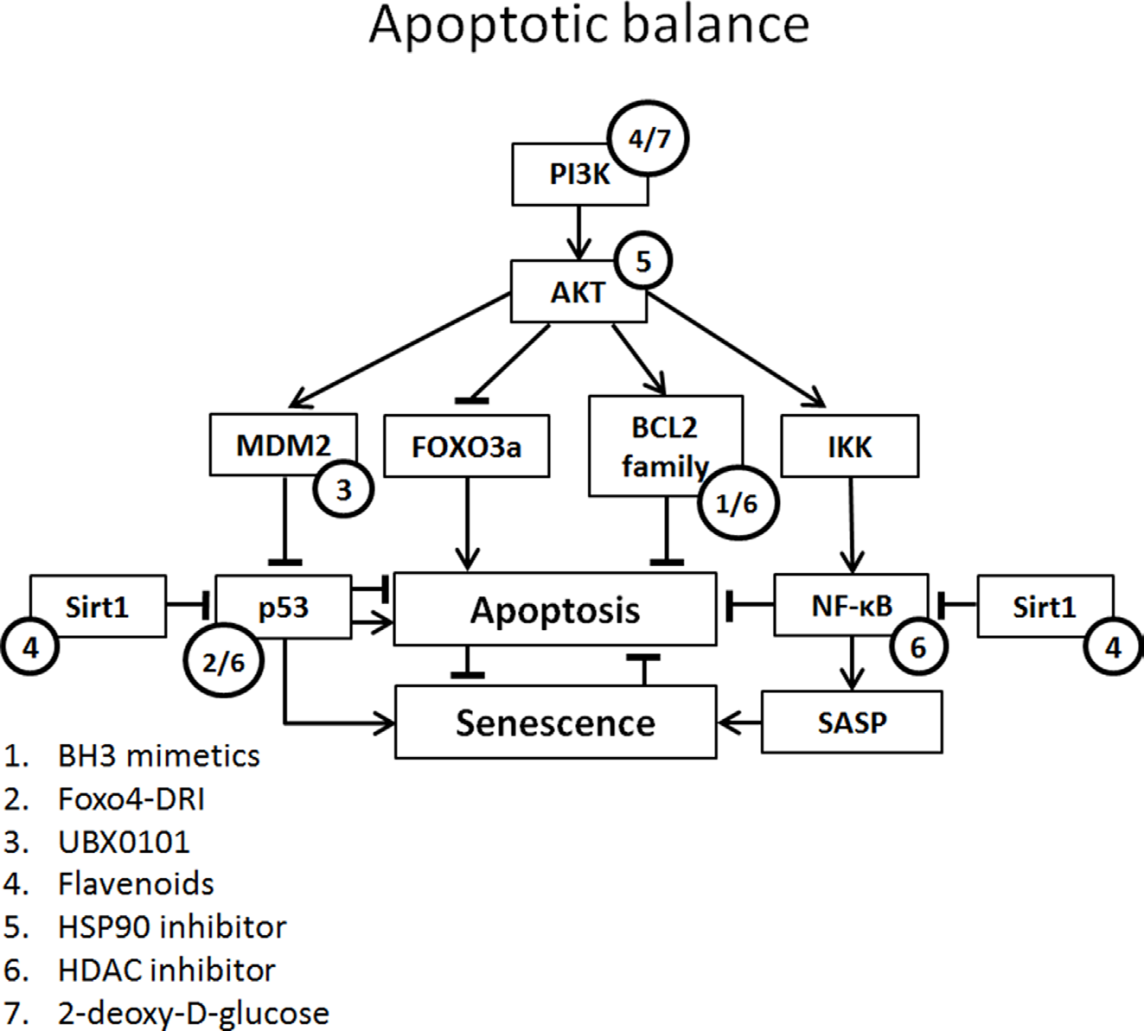
Apoptotic balance of senescent cells with potential targets of senolytics. Apoptotic balance is influenced by a plethora of mediators. Abbreviations: FOXO3, forkhead box O3; IKK, IκB kinase complex.

Therefore, the use of existing antitumor drugs as senolytics has gained interest. Although very few of these drugs have been tested on renal tubular epithelium, many have already been tested on a number of different cell types and tissues both in vivo (**Table 3**) and in vitro (**Table 4**), which may render a first impression of their potential for application in kidney diseases. Below, we therefore review the effects of candidate senolytic drugs on different cell types exposed to various senescence-inducing triggers. For their targets, see **Figure 2**.

**Table 3:** *In vivo* effect of potential senolytic therapy.

Group	Drug	Target	Model	Result	Reference
**BH3 mimetics**	Navitoclax (ABT-263)	BCL-2, BCL-X_L_, BCL-W inhibitor	-Body radiation -Normal aging	-Reduced lung fibrosis -Improved lung tissue elasticity -Improved hematopoietic parameters -Decreased SASP factors	(Chang et al., 2016; Mikawa et al., 2018)
			Induced emphysema (porcine pancreatic elastase)	-Improved lung tissue elasticity	
	ABT-737	BCL-2, BCL-X_L_, BCL-W inhibitor	-Body radiation -p14^ARF^-induced senescence	-Decreased cellular senescence in lungs and skin	(Yosef et al., 2016)
**FOXO4 inhibitor**	FOXO4-DRI	FOXO4-p53 interaction inhibition	-Doxorubicin -Normal aging -Premature aging model	-Decreased doxorubicin-induced body weight and liver toxicity -Improved fur density and responsiveness -Improved kidney function	(Baar et al., 2017)
**UBX0101**	UBX0101	MDM2-p53 pathway	-Post-traumatic osteoarthritis (ACLT transection)	-Reduced pain -Decreased cartilage erosion/thinning -Decreased SASP factors	(Jeon et al., 2017)
**Flavenoids-TKI combination**	Quercetin and Dasatinib	BCL-2 pathway inhibitor (proteasome activator) PI3K and serpine inhibitor Tyrosine kinase inhibitor (EFNB-dependent suppression of apoptosis)	-Natural aging -Single leg radiation -Premature aging model -Atherosclerosis model -Liver steatosis model -Transplant of senescent adipocytes -Pulmonary fibrosis (bleomycin induced)	-Decreased osteoclast numbers -Improved left ventricular ejection fraction -Increased exercise time, distance, and total work to exhaustion -Decreased age-related symptoms -Reduced intimal plaque calcification -Improved vascular relaxation -Reduced hepatic fat deposition -Attenuated decrease of physical function -Attenuated weight reduction -Attenuated lung compliance -Increased distance until exhaustion	(Zhu et al., 2015; Roos et al., 2016; Farr et al., 2017; Ogrodnik et al., 2017; Schafer et al., 2017; Xu et al., 2018)
**Flavenoids**	Fisetin	(hydrophobic groove of) BCL-2	-Natural aging -Premature aging model	-Extension of median and maximal lifespan -Reduced senescent mesenchymal stem/progenitor cells -Reduced SASP factors	(Yousefzadeh et al., 2018)
**HSP90 inhibitors**	17-DMAG	-Inhibits the molecular chaperone HSP90, leading to AKT and ERK destabilization	-Premature aging model	-Reduced age-related symptoms and increased body condition -Reduced kidney p16^Ink4a^ expression	(Fuhrmann-Stroissnigg et al., 2017)

**Table 4:** *In vitro* senolytic effect of tested drugs on different cell lines and different manners of senescence induction.

Group	Drug	Target	Cell line	Induction	Senolytic	Reference
**BH3 mimetics**	Navitoclax (ABT-263)	BCL-2, BCL-X_L_, BCL-W inhibitor	-WI-38	-Radiation -Replicative exhaustion -Oncogenic RAS expression	-Yes -Yes -Yes	(Chang et al., 2016; Zhu et al., 2016; Fuhrmann-Stroissnigg et al., 2017; Pan et al., 2017; Schafer et al., 2017; Grezella et al., 2018)
			-IMR90	-Radiation -Etoposide	-Yes -No	
			-RECs	-Radiation	-Yes	
			-MEFs	-Radiation -Premature aging	-Yes -Yes	
			-HUVECs	-Radiation	-Yes	
			-Human preadipocytes	-Radiation	-No	
			-Human MSCs	-Replicative exhaustion	-Yes	
			-AECIIs primary pneumocytes (C57BL/6J mice)	-Radiation	-Yes	
	Venetoclax (ABT-199)	BCL-2 inhibitor	-WI-38	-Radiation	-Yes, in combination with WEHI-539	(Chang et al., 2016)
			-IMR90	-Etoposide -Oncogenic H-Ras expression	-No -Yes	(Yosef et al., 2016)
	ABT-737	BCL-2, BCL-X_L_, BCL-W inhibitor	-IMR90	-Radiation -Etoposide -Oncogenic H-Ras expression -Replicative exhaustion	-Yes -Yes -Yes -Yes	(Yosef et al., 2016; Baar et al., 2017)
	WEHI-539	BCL-X_L_ inhibitor	-WI-38	-Radiation	-Yes in combination with venetoclax	(Chang et al., 2016)
	TW-37	BCL-2, MCL-1 inhibitor	-IMR90	-Radiation	-Yes	(Zhu et al., 2016)
			-HUVECs	-Radiation	-Yes	
			-Human readipocytes	-Radation	-Yes	
			-MEFs	-Oxidative stress -Replicative exhaustion	-No -No	
	A1331852	BCL-X_L_ inhibitor	-IMR90	-Radiation	-Yes	(Zhu et al., 2017)
			-HUVECs	-Radiation	-Yes	
			-Human preadipocytes	-Radiation	-No	
	A1155463	BCL-X_L_ inhibitor	-IMR90	-Radiation	-Yes	
			-HUVECs	-Radiation	-Yes	
			-Human preadipocytes	-Radiation	-No	
	Obatoclax (GX15-070)	BCL-2, BCL-X_L_, BCL-W inhibitor	-IMR90	-Etoposide -Oncogenic H-Ras expression	-No	(Yosef et al., 2016)
**FOXO4 inhibitor**	FOXO4-DRI	FOXO4-p53 interaction inhibition	-WI-38	-Radiation	-Yes	(Baar et al., 2017)
			-IMR90	-Radiation -Doxorubicin	-Yes -Yes	
			-BJ	-Radiation	-Yes	
**UBX0101**	UBX0101	MDM2/p53	-Primary human chondrocytes	-Osteoarthritis patient	-Yes	(Jeon et al., 2017)
**Flavenoids and TKI**	Quercetin and dasatinib	BCL-2 pathway inhibitor (proteasome activator) PI3K and serpine inhibitor Tyrosine kinase inhibitor (EFNB-dependent suppression of apoptosis)	-IMR90	-Radiation -Etoposide	-Yes/no -Yes	(Zhu et al., 2015; Baar et al., 2017; Fuhrmann-Stroissnigg et al., 2017; Lehmann et al., 2017; Schafer et al., 2017; Geng et al., 2019; Grezella et al., 2018; Xu et al., 2018)
			-Pulmonary mouse ATII cells	-Bleomycin	-Yes	
			-HUVECs	-Radiation -Replicative exhaustion	-Yes -Yes	
			-Human preadipocytes	-Radiation -Replicative exhaustion	-Yes/no -Yes	
			-MEFs	-Radiation -Premature aging	-Yes -Yes	
			-MSCs	-Replicative exhaustion -Premature aging	-No -No	
	Fisetin	BCL-2 pathway inhibitor (hydrophobic groove of BCL-2)	-IMR90	-Etoposide	-No (decreased senescence, no apoptosis)	(Yousefzadeh et al., 2018)
			-HUVECs	-Etoposide	-Yes	
			-MEFs	-Etoposide -Premature aging	-No -No	
			-MSCs	-Replicative exhaustion	-No	(Zhu et al., 2017)
**HSP90 inhibitors **	17-DMAG	Inhibits the molecular chaperone HSP90, leading to AKT and ERK destabilization	-WI-38	-Replicative exhaustion	-Yes	(Fuhrmann-Stroissnigg et al., 2017)
			-IMR90	-Etoposide	-Yes	
			-MEFs	-Premature aging	-Yes	
			-MSC	-Oxidative stress	-Yes	
	Geldanamycin		-MEFs	-Premature aging	-Yes	
**Niacine**	Nicotinamide riboside	Nicotinamide adenine dinucleotide (NAD^+^) increase	-MSCs	-Replicative exhaustion	-No	(Grezella et al., 2018)
**Resveralogues**	Resveratrol	Activation of SIRT1 (NAD-dependent protein deacetylase)	-NHDF (human fibroblasts)	-Replicative exhaustion	-No	(Latorre et al., 2017)
			-HF043 (human dermal fibroblasts)	-Replicative exhaustion	-No	
			-MRC5 (human lung fibroblasts)	-Replicative exhaustion	-No	

Senolytic effect is defined as a selective induction of apoptosis in senescent cells. Cell lines: WI-38, human lung fibroblasts; IMR90, human lung fibroblasts; RECs, human renal epithelial cells; HUVECs, human umbilical vein endothelial cells; BJ, human foreskin fibroblasts; MSC (femoral bone marrow); NHDFs, normal human dermal fibroblasts.

#### BH3 Mimetics

BH3 protein (e.g., BIM, BID, and PUMA) is up-regulated by stress signals and binds with pro-survival BCL-2 protein family. This inhibits the interaction of BCL-2 family protein with BAX and BAK. Released BAX and BAK form oligomers and initiate the caspase cascade by perforating the outer mitochondrial membrane leading to apoptosis, a process circumvented by senescence *via* an increase in BCL-2 family member expression (Adams and Cory, 2018).

BH3 mimetics inhibit the binding of BCL-2 family to BAX and BAK, enabling BAX and BAK to form oligomers and induce apoptosis. There are several BH3 mimetics targeting a different (combination of) BCL-2 family protein(s). One of the more successful being venetoclax (ABT-199; BCL-2 inhibitor), which has been FDA approved for the treatment of refractory chronic lymphocytic leukemia (Adams and Cory, 2018). Although successful in oncology, the *in vitro* senolytic effect of venetoclax in human fibroblasts is variable and dependent on the manner of senescence induction (Chang et al., 2016; Yosef et al., 2016).

A promising and more extensively tested BH3 mimetic is navitoclax (ABT-263; BCL-2, BCL-XL, and BCL-w inhibitor). In several studies, navitoclax induced a senolytic effect in different human and mouse cells, including renal epithelial cells (Chang et al., 2016; Zhu et al., 2016; Fuhrmann-Stroissnigg et al., 2017; Kim et al., 2017; Pan et al., 2017; Grezella et al., 2018). However, in human MSCs, navitoclax showed only low selectivity for senescent cells compared to non-senescent cells and no senolytic effect was observed in human preadipocytes (Zhu et al., 2016; Grezella et al., 2018). Navitoclax clears senescent cells and reduces SASP in old and irradiated mice. Furthermore, treatment of mice with navitoclax results in improved myeloid function and reversed pulmonary fibrosis (Chang et al., 2016; Pan et al., 2017).

The BH3 mimetic ABT-737 (BCL-2 and BCL-XL inhibitor) causes a selective reduction in senescent human and mouse fibroblasts in several models of senescence (etoposide, H-ras, replicative), but a lesser reduction is seen in radiation-induced senescent fibroblasts (Yosef et al., 2016; Baar et al., 2017). The *in vivo* application of ABT-737 results in a significant reduction of senescent cells, i.e., in the basal layer of skin epidermis after radiation and p14^ARF^-induced senescence (Yosef et al., 2016).

Several other BH3 mimetics have been studied in the context of senescence. The BCL-X_L_ inhibitors A1331852 and A1155463 both selectively reduce the viability of radiation-induced senescent HUVECs and IMR90s compared to non-senescent cells. Like navitoclax, no selective reduction of senescent cell viability is seen in preadipocytes (Zhu et al., 2017). TW-37 (BCL-2 and MCL-1 inhibitor) is less senolytic compared to navitoclax in radiation-induced senescent endothelial cells and fibroblasts (Zhu et al., 2016). No senolytic effect is observed in preadipocytes. WEHI-539 (BCL-X_L_ inhibitor) shows no senolytic effect in radiation-induced senescent WI-38 cells (Chang et al., 2016). Obatoclax (BCL-2, BCL-X_L_, and BCL-W inhibitor) induces a significant reduction in both control cell and senescent human fibroblasts (Yosef et al., 2016).

#### Flavenoids

##### Quercetin

Quercetin is a flavonol. It belongs to the polyphenols and is subclassified as a flavonoid. It is found in fruits and vegetables and the average daily consumption amounts to approximately 10 mg/day. Quercetin has an anti-oxidative effect, targeting ROS and reactive nitrogen species (ROS). Apart from this direct anti-oxidative effect, quercetin has an indirect anti-oxidative effect *via* activation of the nuclear factor (erythroid-derived 2)-like 2 (Nrf2) and paraoxonase 2 (PON2). Both pathways have an antioxidant effect (Costa et al., 2016). Interestingly, quercetin activates sirtuin 1 (Sirt1), a nicotinamide dinucleotide-dependent deacetylase that has a renoprotective effect, mediated by the deacetylation of p53, improving mitochondrial function and decreasing NF-κB, resulting in decreased fibrogenesis (Wakino et al., 2015).

Quercetin has most often been studied in combination with the tyrosine kinase inhibitor dasatinib. *In vitro*, this combination selectively reduces senescent human endothelial cells and mouse fibroblasts while being less effective in pre-adipocytes and human lung fibroblasts (Zhu et al., 2015; Baar et al., 2017; Fuhrmann-Stroissnigg et al., 2017; Schafer et al., 2017; Grezella et al., 2018). In replicative senescent human MSCs, no clear senolytic effect was observed, whereas in Werner syndrome and in a Hutchinson-Gilford progeria model quercetin (without dasatinib) caused a decrease in senescence markers in human MSCs (Zhu et al., 2015; Geng et al., 2019). Treatment with quercetin and dasatinib of primary human adipose tissue from obese patients resulted in the decreased expression of senescence markers and reduced SASP production (Xu et al., 2018).

Quercetin and the quercetin-dasatinib combination have been applied in several mouse models, including naturally and accelerated aging mice, obesity-induced metabolic dysfunction, single-leg radiation, atherosclerosis model, liver steatosis, bleomycin-induced pulmonary injury, and transplantation of senescent cells. Combined treatment with quercetin and dasatinib of mice with metabolic syndrome not only resulted in improved glucose metabolism but also resulted in a decreased albuminuria (Palmer et al., 2019). In naturally aged mice, combined treatment with quercetin and dasatinib resulted in decreased cell senescence in fat and liver, improved left ventricular ejection fraction, decreased senescence in endothelial and smooth muscle layers, reduced physical dysfunction, reduced SASP expression, and improved bone microarchitecture (Zhu et al., 2015; Roos et al., 2016; Farr et al., 2017; Xu et al., 2018). Treatment of mice results in a remarkable improvement of several aging-related parameters, such as condition, muscle function, and coordination (Zhu et al., 2015).

##### Fisetin

Fisetin is another member of the flavonoid family and is also found in fruits and vegetables. Fisetin targets a plethora of signaling pathways including PI3K/AKT, NF-κB, p38 mitogen-activated protein kinase (MAPK), and BCL-2/BCL-X_L_ (Sundarraj et al., 2018). Interestingly, fisetin has been shown to be beneficial in cisplatin-induced AKI (Sahu et al., 2014).

*In vitro*, fisetin reduces (etoposide-induced) senescent cells in human and mouse cells but not in replicative senescent human fibroblasts. Interestingly, in mouse fibroblasts, fisetin does not induce apoptosis, suggesting that the effect is not senolytic in these cells but that fisetin alleviates the cell cycle arrest (Yousefzadeh et al., 2018).

In naturally aged mice (22–24 months) and a XFE progeria model (p16^+/Luc^; Ercc1^-/Δ^), fisetin treatment results in an extension of median and maximal lifespan and a decrease in cellular senescence in liver, kidney, fat, spleen, and peripheral CD3^+^ T cells and a decrease in circulating SASP factors (Yousefzadeh et al., 2018).

#### p53-Mediated Senolytics

##### FOXO4

The FOXO4 D-retro inverso (DRI) peptide targets the FOXO4-p53 axis, leading to an increase in active p53 in senescent cells (release from DNA-SCARS). FOXO4 DRI increases the mouse quality of life (fur and activity) in naturally old and genetically aged models of senescence in mice. In a mouse model of folic acid-induced kidney damage, a significant reduction in senescent tubular cells was seen, although no reduction in renal fibrosis, cellular infiltrate, and tubular damage was seen (Jin et al., 2019). Moreover, in the Xdp^TTD/TTD^ p16:3MR mouse model of accelerated aging, FOXO4 DRI treatment was associated with preserved kidney function (Baar et al., 2017). FOXO4 DRI treatment of senescent human fibroblasts resulted in a selective reduction of senescent cells compared to non-senescent cells (Baar et al., 2017).

##### UBX0101

UBX0101 is a small-molecule inhibitor targeting MDM2/p53 interaction. Treatment of primary human chondrocytes isolated from osteoarthritis patients with UBX0101 showed a senolytic effect of UBX0101 and reduced SASP expression. In a post-traumatic osteoarthritis mouse model (ACTL transection), treated mice showed reduced pain and reduced articular cartilage erosion and less senescent markers in the cartilage. The improvement in physical function lasted 84 days (Jeon et al., 2017). A phase 1 clinical trial regarding osteoarthritis is ongoing (ClinicalTrials.gov Identifier: NCT03513016).

#### Heat Shock Protein 90 Inhibitors

Heat shock protein 90 (HSP90 is a molecular chaperone that is especially transcribed during cellular stress, aiding protein stabilization and preventing protein misfolding and aggregation. The so-called client proteins of HSP90 include transcription factors (e.g., p53), kinases (e.g., CDK4), eNOS, TERT, and mitochondrial proteins. In tumor cells, HSP90 has an anti-apoptotic effect mediated by mTOR, NF-κB, and FOXO3A; furthermore, it leads to AKT stabilization mitigating apoptosis (Taipale et al., 2010; Fuhrmann-Stroissnigg et al., 2017).

The senolytic potential of HSP90 inhibitors was discovered in a screening assay for senotherapeutics using Ercc1^-/-^ mouse embryonic fibroblasts (MEFs). Several possible senolytic agents involved in regulating autophagy were analyzed and treatment with the HSP90 inhibitors 17-DMAG and geldanamycin resulted in the strongest senolytic effect. The senolytic effects of 17-DMAG are also seen in senescent human MSCs and fibroblasts (Fuhrmann-Stroissnigg et al., 2017). Furthermore, progeroid mice (Ercc1^-/Δ^) treated with 17-DMAG showed reduced age-related symptoms and increased overall condition (Fuhrmann-Stroissnigg et al., 2017).

#### Panobinostat

Panobinostat (LBH-589) is a histone deacetylase (HDAC) inhibitor and is FDA and European Medicines Agency approved for the treatment of refractory multiple myeloma (Tzogani et al., 2018). *In vivo* studies with HDAC inhibitors show attenuation of renal inflammation and fibrosis in several animal models for kidney disease (Brilli et al., 2013; Van Beneden, et al., 2013). HDAC causes the deacetylation of lysine residues of histone tails, promoting the interaction of histones and DNA, inhibiting mRNA transcription. Other targets of HDAC besides the histones are E2F, p53, and NF-κB. Inhibitors of HDAC increase p21 transcription levels and result in the acetylation of p53, thereby promoting cell cycle arrest (Yoon and Eom, 2016). However, HDAC inhibitors also have a marked effect on the apoptotic balance in the cell, increasing transcription of proapoptotic proteins Bax, Bak, Bim, Bad, Noxa, Puma, Bid, and Apaf1 and decreasing expression of BCL-2, BCL-XL, Mcl-1, and survivin (Marchion and Münster, 2007). This pro-apoptotic effect could explain the senolytic effect seen in senescent non-small cell lung cancer and head and neck squamous cancer after chemotherapy (Samaraweera et al., 2017).

#### (2DG)



2DG is a glucose analog that competitively inhibits the uptake of glucose by GLUTs. Once inside the cell, 2DG inhibits ATP production, in turn activating AMPK and furthermore leading to cell cycle arrest, decreased cell growth, increased autophagy, and cell death (Zhang et al., 2014). 2DG shows a senolytic effect on senescent vascular smooth muscle cells. The potential senolytic effect of 2DG relies on the increased metabolic activity (glucose consumption) of senescent cells (Gardner et al., 2015).

To summarize, the identified senolytics, senomorphics, and SASP modulators show varying results on *in vivo* and *in vitro* models for aging and disease. This variance is highly dependent on the cell type and manner of senescence induction. It is therefore important to investigate the senotherapeutic drugs in the cell type of interest using the appropriate induction of senescence. The senotherapies tested in *in vivo* kidney disease models show promising signs of kidney function preservation and reduced kidney fibrosis.

## Targeted Therapy

Although senolytic therapy is potentially beneficial in reversing age-related diseases, off-target effects might occur. For instance, loss of the beneficial role of cellular senescence in cutaneous wound healing and in the prevention of fibrosis upon liver injury (Krizhanovsky et al., 2008; Demaria et al., 2014). Furthermore, several potent senolytic agents such as navitoclax and ABT-737 show major systemic side effects such as thrombocytopenia and neutropenia when administered systemically (Wilson et al., 2010; Kipps et al., 2015).

Other hurdles in senolytic therapy for kidney disease are the first-pass hepatic clearing of molecules reducing bioavailability and thus kidney exposure and the rapid passaging of molecules through the kidney, leaving little time for the molecules to have effect.

These hurdles can in part be overcome by the intermittent administration of the senolytic drugs while still achieving the desired effect. Moreover, a targeted delivery of senolytic compounds to senescent renal cells would allow for a decrease in systemic exposure and toxicity.

Targeted accumulation of senolytic agents in the kidney might be achieved using nanomedicines (i.e., nanoparticulate carriers) such as conjugates and liposomes. The delivery of therapeutic of such functionalized compounds should enable high enough drug concentrations where needed.

Major methods of targeting proximal renal tubular cells are the use of protein- or peptide-based carriers and the use of nanoparticles (Liu et al., 2019). Protein- or peptide-based carriers consist of a low molecular weight protein (LMWP; e.g., lysozyme and immunoglobulins) to which the drug is linked (Zhou et al., 2014). In case of proximal tubular epithelium, the LMWP has a high affinity for tubular cell membrane receptors such as low-density lipoprotein receptor-related protein 2 (megalin and cubilin) (Christensen et al., 2012). Upon binding to the membrane receptor, the drug and LMWP are internalized *via* endocytosis. After degradation of the bond between drug and LMWP in the lysosome, the drug enters the cytoplasm (Nasiri et al., 2018).

Nanoparticles are engineered organic or inorganic carriers typically smaller than 150 nm, designed to deliver drugs to specific organs or cell types, depending on the nanoparticle’s characteristics. Depending on the desired target, nanoparticles can be designed varying in size, shape, charge, and composition. Furthermore, the surface of the nanoparticle can be coated with ligands (e.g., immunoglobulins) targeting specific cell types (Kamaly et al., 2016). To pass the glomerular filtration barrier and reach luminal surface of the tubular epithelium, nanoparticles need to be smaller than 5 to 7 nm. A positive charge facilitates, whereas a negative charge hampers passage (Kamaly et al., 2016). Interestingly, however, much larger mesoscale nanoparticles of 400 nm were found to selectively target proximal epithelial cells possibly by transcytosis across capillaries and endocytosis into the epithelium (Williams et al., 2015).

Examples of drug targeting to the proximal tubular epithelium include the conjugation of imatinib — platelet-derived growth factor receptor kinase inhibitor—to lysozyme *via* a platinum (II)-based Universal Linkage System (ULS). This resulted in a bioavailability of 100% when to imatinib-ULS-lysozyme was administered either intravenously or in the intraperitoneal cavity and a decreased exposure of other organs to imatinib in mice (Dolman et al., 2012).

## Challenges and Opportunities

The timing of the senolytic treatment of CKD is of great importance. Due to the limited regenerative potential of the kidney in CKD, treatment with senolytics would seem most beneficial in the early stages of CKD when the number of senescent cells is still limited and little progression of fibrosis has occurred. The identification of biomarkers to identify this “window of opportunity” will be of great help to make the best use of senolytics.

Because the actual disease burden of early CKD is relatively limited, (potential) side effects of senolytics will constitute major hurdles to the application of such drugs at this stage of disease progression. Therefore, it will be essential to keep side effects to a minimum.

For this, it is important to realize that senescent cells can express specific surface markers such as NKG2D ligands (MIC and RAET1/ULBP related), identifying them for elimination by surveilling immune cells. The fact that perforin KO in mice leads to the accumulation of senescent cells suggests that targeted intracellular delivery of granzymes might constitute an effective approach for novel senolytic therapies (Ovadya et al., 2018). Senescence-induced surface markers might also be explored for the targeted delivery of senolytic drugs in general.

Research regarding senescence in the kidney has pointed to the proximal tubular epithelium as the culprit, and the removal of senescent tubular epithelial cells is therefore a promising approach to the attenuation of fibrosis in CKD, an otherwise untreatable and progressive disease (Baar et al., 2017). Due to the specific nature of proximal tubular epithelium, several specific targeting options are available, by which therapeutic drug efficacy can be potentiated and side effects can be reduced. Repurposing senolytic drugs that have been tested in clinical trials for other, mostly oncological, indications by functionalization for targeted delivery is a promising method to make a fast translation to clinical nephrology practice.

## Author Contributions

SK and FV performed the literature search and wrote the manuscript. TN, RG, and LF reviewed and edited the manuscript.

## Funding

This work is supported by the Dutch Kidney Foundation (Kolff Grant 17OKG20 and Consortium Grant CP18.05).

## Conflict of Interest Statement

The authors declare that the research was conducted in the absence of any commercial or financial relationships that could be construed as a potential conflict of interest.
